# Complexity Matching: Restoring the Complexity of Locomotion in Older People Through Arm-in-Arm Walking

**DOI:** 10.3389/fphys.2018.01766

**Published:** 2018-12-04

**Authors:** Zainy M. H. Almurad, Clément Roume, Hubert Blain, Didier Delignières

**Affiliations:** ^1^Euromov, University of Montpellier, Montpellier, France; ^2^College of Physical Education, University of Mosul, Mosul, Iraq; ^3^Montpellier University Hospital, Montpellier, France

**Keywords:** complexity matching, restoration of complexity, interpersonal coordination, arm-in-arm walking, rehabilitation

## Abstract

The complexity matching effect refers to a maximization of information exchange, when interacting systems share similar complexities. Additionally, interacting systems tend to attune their complexities in order to enhance their coordination. This effect has been observed in a number of synchronization experiments, and interpreted as a transfer of multifractality between systems. Finally, it has been shown that when two systems of different complexity levels interact, this transfer of multifractality operates from the most complex system to the less complex, yielding an increase of complexity in the latter. This theoretical framework inspired the present experiment that tested the possible restoration of complexity in older people. In young and healthy participants, walking is known to present 1/*f* fluctuations, reflecting the complexity of the locomotion system, providing walkers with both stability and adaptability. In contrast walking tends to present a more disordered dynamics in older people, and this whitening was shown to correlate with fall propensity. We hypothesized that if an aged participant walked in close synchrony with a young companion, the complexity matching effect should result in the restoration of complexity in the former. Older participants were involved in a prolonged training program of synchronized walking, with a young experimenter. Synchronization within the dyads was dominated by complexity matching. We observed a restoration of complexity in participants after 3 weeks, and this effect was persistent 2 weeks after the end of the training session. This work presents the first demonstration of a restoration of complexity in deficient systems.

## Introduction

Complexity appears a key concept for the understanding of the perennial functioning of biological systems. By definition, a complex system is composed of a large number of infinitely entangled elements (Delignières and Marmelat, 2012). In such a system, interactions between components are more important than components themselves, a feature that Van Orden et al. (2003) referred to as *interaction-dominant* dynamics.

Such a system, characterized by a myriad of components and sub-systems, and by a rich connectivity, could lose its complexity in two opposite ways: either by a decrease of the density of interactions between its components, or by the emergence of salient components that tend to dominate the overall dynamics. In the first case the system derives toward randomness and disorder, in the second toward rigidity. From this point of view complexity may be conceived as an optimal compromise between order and disorder (Delignières and Marmelat, 2012). Complexity represents an essential feature for living systems, providing them with both robustness (the capability to maintain a perennial functioning despite environmental perturbations) and adaptability (the capability to adapt to environmental changes). These relationships between complexity, robustness, adaptability and health were nicely illustrated by Goldberger et al. (2002a) in the domain of heart diseases.

The experimental approach to complexity has been favored by the hypothesis that links the complexity of systems and the correlation properties of the time series they produce, and the development of related fractal analysis methods, and especially the Detrended Fluctuation Analysis (Peng et al., 1995). A complex system is supposed to produce long-range correlated series (1/*f* fluctuations), and the assessment of correlation properties in the series produced by a system allows determining the possible alterations of complexity, either toward disorder (in which case correlations tend to extinguish in the series) or toward rigid order (in which case correlations tend to increase).

This interest for complexity was particularly developed in the research on aging. According to Lipsitz and Goldberger (1992), aging could be defined by a progressive loss of complexity in the dynamics of physiologic systems. This hypothesis has been developed in a number of subsequent papers (Goldberger et al., 2002a,b; Vaillancourt and Newell, 2002; Sleimen-Malkoun et al., 2014). Of special interest for the present work, Hausdorff and collaborators showed that the series of stride durations, during walking, presented a typical structure over time, characterized by the presence of long-range correlation (Hausdorff et al., 1995, 1996, 2001). They also showed that these scaling properties were altered in aged participants, and also in patients suffering from Huntington’s disease (Hausdorff et al., 1997). In those cases the fractal structure tended to disappear and stride dynamics derived toward randomness. Additionally, they showed that the loss of complexity in stride duration series correlated with the propensity to fall. The main question we address in the present paper is the following: could it be possible to restore complexity in older people, and especially in the locomotion system?

The hypothesis that sustains the present work is based on the concept of *complexity matching*, initially introduced by West et al. (2008). The complexity matching effect refers to the maximization of information exchange when interacting systems share similar complexities. Aquino et al. (2011) interpreted this effect as a kind of “1/*f* resonance” between systems.

Marmelat and Delignières (2012) proposed a working conjecture stating that interacting systems tend to match their complexities in order to enhance their synchronization. This attunement of complexities has been observed in a number of synchronization experiments (Stephen et al., 2008; Marmelat and Delignières, 2012; Abney et al., 2014; Delignières and Marmelat, 2014; Coey et al., 2016; Almurad et al., 2017). Mahmoodi et al. (2018) emphasized the role of crucial events, which play a fundamental role in the transport of information between complex networks. Crucial events are generated by the processes of self-organization, and the series of waiting times separating successive crucial events presents 1/*f* properties. The transfer of information between systems is accomplished through the matching of the fractal properties of the series of waiting time of the interacting systems. Moreover Mahmoodi et al. (2018), adopting a theoretical approach based on subordination theory, showed that when two systems of different complexity levels interact, the most complex system attracts the less complex, yielding an increase of complexity in the latter (see also Mahmoodi et al., 2017). This theoretical result is fundamental in the present work.

The very first experimental approaches to complexity matching considered that a close correlation between the mono-fractal exponents characterizing the two synchronized systems could represent a satisfactory evidence for complexity matching (Stephen et al., 2008; Marmelat and Delignières, 2012; Delignières and Marmelat, 2014; Marmelat et al., 2014). However, Delignières et al. (2016) considered that this correlation between scaling exponents does not represent an unequivocal evidence for complexity matching. They proposed to distinguish between simple *statistical matching* (characterized by a convergence of scaling exponents) and *genuine complexity matching* (defined as the attunement of complexities). Indeed, recent studies showed that the correlation between fractal exponents could just be the consequence of local corrective processes (Torre et al., 2013; Delignières and Marmelat, 2014; Fine et al., 2015). For example Delignières and Marmelat (2014), in an experiment where participants had to walk in synchrony with fractal metronomes, evidenced a close correlation between the scaling exponents of the stride intervals series produced by participants and those of the corresponding metronomes. However, they showed that a model based on a stride-to-stride correction of asynchronies satisfactorily reproduced this statistical matching. They concluded that synchronization with a fractal metronome could be essentially based on short-term correction processes, and that the matching of scaling exponents could just result from these local corrections.

Delignières et al. (2016) introduced a new method for distinguishing between local corrective processes and genuine complexity matching. They proposed to analyze statistical matching on the basis of a multifractal approach, rather than on the monofractal level. Indeed, the multifractal approach allows for a more detailed picture of complexity in the series. More fundamentally, some authors argued that the tailoring of fluctuations typically observed in complexity matching should be considered the product of multifractality (Stephen and Dixon, 2011), and Mahmoodi et al. (2017) considered complexity matching as a transfer of multifractality between system.

Multifractal processes, compared to monofractal series, exhibit more complex fluctuations and cannot be fully characterized by a single exponent: Subsets with small and large fluctuations scale differently, and their complete description necessitates a set of scaling exponents (Podobnik and Stanley, 2008). Delignières et al. (2016) proposed to evaluate statistical matching through point-by-point correlation functions, computed between the sets of exponents that characterize both coordinated series. They assessed these correlations over multiple ranges of intervals, in first over the largest range (e.g., from 8 to *N*/2, *N* representing the length of the series), and then over more and more narrow ranges, with a progressive exclusion of the shortest intervals (i.e., from 16 to *N*/2, from 32 to *N*/2, and then from 64 to *N*/2). They supposed that from the moment that the systems are effectively synchronized, and whatever the way this synchronization is achieved, one could expect to find close correlations when only long-term intervals are considered (i.e., from 64 to *N*). If synchronization is mainly based on local corrections, correlations should dramatically decrease when intervals of shorter durations are considered, because such local corrections are necessarily approximate between unpredictable systems (Stephen et al., 2008). In contrast, for genuine complexity matching, synchronization emerges from interactions across multiple scales, and one should observe close correlations, even when the entire range of available intervals is considered (Delignières et al., 2016).

Almurad et al. (2017) and Roume et al. (2018) proposed another method, the Windowed detrended cross-correlation analysis (WDCC), based on the analysis of cross-correlations between the series produced by the two systems. In this method, the series is divided into short intervals of 15 data points, detrended within each interval, and the cross-correlation function, from lag −10 to lag 10, is computed within each interval. An averaged windowed detrended cross-correlation function is then obtained by averaging over all intervals. Windowing allows focusing on local synchronization processes, and detrending controls the effect of local trends, which tends to spuriously inflate cross-correlations. Similar approaches were already used in other papers, albeit differing in some methodological settings (Konvalinka et al., 2010; Delignières and Marmelat, 2014; Coey et al., 2016; Den Hartigh et al., 2018).

Windowed detrended cross-correlation analysis allows distinguishing complexity matching from synchronization processes based on discrete asynchronies corrections: in the first case the cross-correlation function presents a positive peak at lag 0, whereas in the second case one obtains positive peaks at lags −1 and 1, and a negative peak at lag 0 (Konvalinka et al., 2010; Almurad et al., 2017; Roume et al., 2018). Additionally, complexity matching seems characterized by quite moderate levels of lag 0 cross-correlation, in contrast with those expected in continuous coupling models (Delignières and Marmelat, 2014; Coey et al., 2016).

Almurad et al. (2017) used these two methods for clarifying the nature of synchronization in side-by-side walking. In this experiment the authors analyzed stride series collected in three conditions: independent, side-by-side, and arm-in-arm walking. They evidenced clear signatures of complexity matching in the two last conditions: In both cases the correlation functions between multi-fractal spectra remained significant, whatever the range of intervals considered, and the WDCC functions showed a positive peak at lag 0. Additionally, this experiment showed that complexity matching was more intense in arm-in-arm than in side-by-side walking.

The hypotheses that are tested in the present work derive from the preceding considerations. Our goal was to investigate the possible restoration of complexity in deficient systems. We supposed, as indicated by Hausdorff et al. (1997), that aging should result in a decrease of the complexity of locomotion, as compared with young and healthy persons.

(1) If an older person is invited to walk in synchrony, arm-in-arm with a healthy partner, we should observe a complexity matching effect within the dyad.
(2) Considering the asymmetry of complexities, complexity matching should result in an increase of complexity in the older person.
(3) A prolonged training of walking in synchrony with healthy partners should induce a perennial restoration of complexity in older persons.

## Materials and Methods

### Participants

Twenty-four participants (7 male and 17 female, mean age: 72.46 ± 4.96 years) were involved in the experiment. They were recruited in local retiree associations, and could be considered as presenting a normal aging. They were free from disease that could affect gait, including any neurological, musculoskeletal, cardiovascular, or respiratory disorders, and had no history of falls. They were randomly assigned to two groups: an experimental group (*N* = 12, 2 male and 10 female, mean age: 72.83 ± 6.01 years, mean weight: 64.25 ± 10.89 kg, mean height: 162.92 ± 6.02 cm), and a control group (*N* = 12, 5 male and 7 female, mean age: 72.08 ± 3.87 years, mean weight: 69.91 ± 8.63 kg, mean height: 166.50 ± 10.39 cm). All work was conducted in accordance with the 1964 Declaration of Helsinki, and was approved by the Euromov International Review Board (n°1610B). Participants signed an informed consent and were not paid for their participation.

### Experimental Procedure

The experiment was performed around an indoor running track (circumference 200 m). Participants were submitted to a walking training during four consecutive weeks, herein noted as week 1, 2, 3, and 4. Each week comprised three training sessions, performed on Monday, Wednesday, and Friday.

Each week, the Monday session began with a *solo sequence*, during which the participant was instructed to walk individually around the track, as regularly as possible, at his/her preferred speed, for 15 min. The aim of this solo sequence was to assess the complexity of the stride duration series produced by the participant. This solo sequence was performed at the beginning of each week, in order to avoid any effect of fatigue.

Then participants performed during each week three *duo sequences* in the Monday session and four in the Wednesday and Friday sessions. During these sequences, they were invited to walk with the experimenter, for 15 min. All participants walked with the same experimenter (female, 46 years). This methodological choice was motivated by the aim of standardizing experimental conditions among participants. In the experimental group, the participant walked arm-in-arm with the experimenter, and was explicitly instructed to synchronize its steps with those of the experimenter during the whole trial. In the control group, the participant and the experimenter walked together, without physical contact, and without any instruction of synchronization. Note that this control condition cannot be assimilated to the side-by-side condition used by Almurad et al. (2017), in which participants were explicitly instructed to synchronize heel strikes. In both groups, the experimenter was instructed to adapt her velocity to that spontaneously adopted by the participant.

Participants had a resting period of at least 10 min between two successive sequences. Each participant performed 44 duo sequences during the whole training program (i.e., 11 walking hours, approximately 67 km). Note that all participants performed approximately the same amount of walk (in terms of duration). The experimental and control groups differed only by the imposed synchronization with the experimenter.

Finally, a solo sequence (post-test) was performed 2 weeks after the end of the training program (i.e., in week 7).

### Data Collection

Data were recorded with two force sensitive resistors (FSR), integrated in soles at heel level. These sensors where wired connected to a Schmitt trigger (LM 393AN), a signal conditioning device that digitally shape the analogic signal of FSR sensors. This device removes noise from the original signal and turns the FSR sensors in on/off switches. The output of the Schmitt trigger was connected to the GPIO interface of a Raspberry Pi model A+. Then, a Wi-Fi dongle (EDIMAX EW7811Un) was plugged in the USB port of the Raspberry and configured as a Hotspot, allowing to launch and remote the device with another. The Schmitt trigger, the Raspberry Pi and a battery (2000 mAh) where packed in a small box entering in a waist bag that was wear on the belt by the participants.

On the software side, the Raspberry Pi was powered by the 2016 February 9th version of the Raspbian distribution. To retrieve the data we wrote a script in Python 3 language, using the internal clock of the Raspberry to time each heel strike, and then to compute stride durations series.

### Statistical Analyses

In the present paper we focused on the series of right stride durations. The raw series comprised 700 to 1300 data points. Fractal analyses are known to be highly sensitive to the presence of local trends in the series, which tend to spuriously increase the assessed level of long-range correlation. In the present experiment, such local trends are related to transient periods of acceleration or deceleration. These local trends are essentially present in the first part of the series, where participants seek for their most comfortable velocity, and at the end of the sequences, essentially due to fatigue, or boredom. The corresponding segments were deleted before analysis.

For solo sequences the resulting series had an average length of 924 points (+/− 148, max = 1257, min = 448), and for duo sequences 963 points (+/−64, max = 1198, min = 397). One could consider that most series satisfied the minimal length required for a valid application of fractal analyses (Delignieres et al., 2006).

We assessed the complexity of each series with the Detrended Fluctuation Analysis (Peng et al., 1994). In the application of DFA, we used intervals ranging from 10 to *N*/2 (*N* representing the length of the series). We applied the evenly spaced algorithm proposed by Almurad and Delignières (2016), which was shown to significantly enhance the accuracy of the original method.

In order to assess the effect of training on the complexity of series in solo sequences, we used a two-way ANOVA 2 (group) X 5 (week), with repeated measurement on the second factor (including the four training weeks and the post-test). Probabilities were adjusted by the Huyn-Feld procedure.

The analysis of synchronization during duo sequences was performed using the methods proposed by Delignières et al. (2016), Almurad et al. (2017), and Roume et al. (2018). We first analyzed the multifractal signature proposed by Delignières et al. (2016): The series were analyzed by means of the *Multifractal Detrended Fluctuation analysis* (MF-DFA, Kantelhardt et al., 2002). MF-DFA was successively applied considering four different ranges of intervals in the series: from 8 to *N*/2, 16 to *N*/2, 32 to *N*/2, and 64 to *N*/2. We used *q*-values ranging from −15 to 15, by steps of 1. The generalized Hurst exponents were then converted into the classical multifractal formalism (Kantelhardt et al., 2002), yielding to the singularity spectra, relating the fractal dimension *f*(α) to the Hölder exponents α(*q*). Finally, we computed for each *q*-value the correlation between the individual Hölder exponents characterizing the two coordinated systems, α_1_(*q*) and α_2_(*q*), respectively, yielding a correlation function *r*(*q*). Whatever the way synchronization was achieved, a correlation function close to 1 is expected, for all *q*-values, when only the largest intervals were considered (i.e., from 64 to *N*/2). When coordination was based on complexity matching, the increase of the range of considered intervals should have a negligible impact on *r*(*q*). In contrast, if coordination was based on discrete asynchronies corrections, a decrease in correlations should be observed.

We then computed for each dyad WDCC functions. We used the sliding version of WDCC, proposed by Roume et al. (2018). In this method, the cross-correlation function is computed over the first available interval, from lag −10 to lag 10. We used intervals of 15 points, and data are linearly detrended within each interval before the computation of cross-correlations. The interval is then lagged by one point, and a second cross-correlation function is computed. This process is repeated up to the last available interval. Finally the cross-correlation functions are point-by-point averaged (for a more detailed presentation of the method, see Roume et al., 2018). As WDDC uses very narrow windows and controls for linear trends, significant correlations are not expected, considering the classical Bravais-Pearson’s test. WDCC just reveals local *traces* of the original correlations, and we are essentially interested by the signs of the average WDCC coefficients, rather than by their statistical significance. Therefore we tested the signs of cross-correlation coefficients with two-tailed location *t*-tests, comparing the obtained values to zero (Roume et al., 2018).

## Results

**FIGURE 1:**
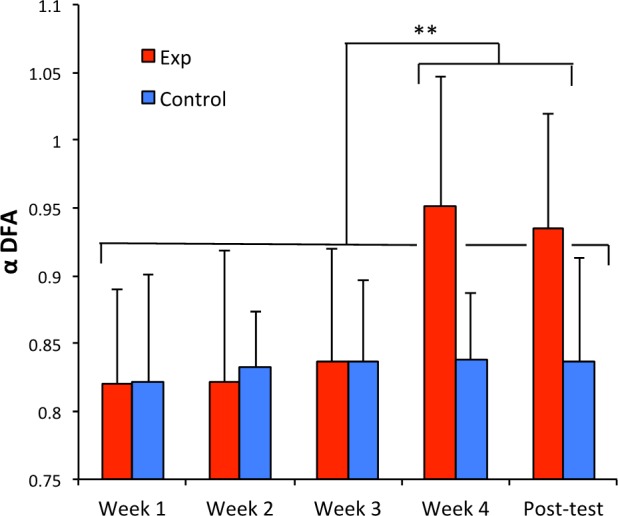
Average α-DFA exponents computed for participants in solo sequences (red: experimental group, blue: control group), over the four training weeks and the post-test. Error bars represent standard deviation. ^∗∗^*p* < 0.01.

We present in Figure 1 the evolution of the average α-DFA exponents computed for participants in solo sequences, for the two groups, over the 4 training weeks and the post-test. The ANOVA revealed a significant interaction effect between Group and Week [*F*(4,88) = 5.084, *p* = 0.001, partial η^2^ = 0.19]. The main effect of Week was also significant [*F*(4,88) = 6.44, *p* = 0.00014, partial η^2^ = 0.23]. A Fisher LSD *post hoc* test showed a significant difference between, on the one hand, the average α-DFA obtained in the experimental group during the fourth week and the post-test, and on the other hand the entire set of other average results.

**FIGURE 2:**
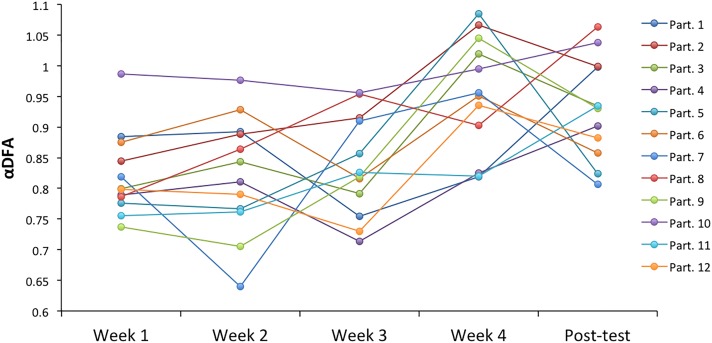
Individual α-DFA exponents in solo sequences, over the 4 training weeks and the post-test, for the 12 participants of the experimental group.

We report in Figure 2 the evolution of individual α-DFA exponents, obtained in solo sequences over the four training weeks and the post-test, for the participants of the experimental group. A detailed examination of this graph reveals some inter-individual differences in the evolution of the scaling parameter. The increase of α exponent at the beginning of the fourth week appeared clearly for 6 participants (# 5, 2, 3, 5, 6, 9, 12), but it occurred early (at the beginning of the third week) for participants 7 and 8. Participant 4 presented at the beginning of the experiment a α exponent close to 1.0, and in that case the protocol had no noticeable effect. One could also note the contrasted evolutions of the exponents between the fourth week and the post-test, with a mix of increases and decreases among participants.

**FIGURE 3:**
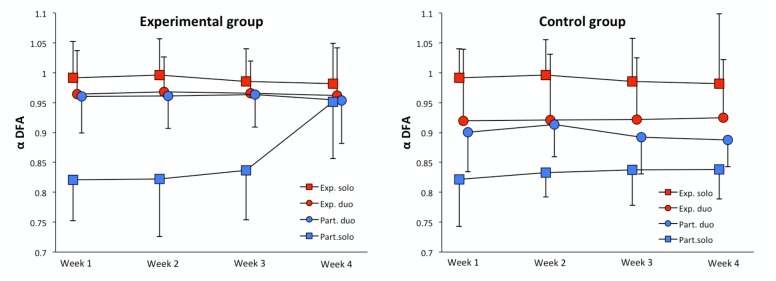
Evolution of the average α-DFA exponent during the 4 weeks (red: experimenter, blue: participants, squares: solo sequences, circles: duo sequences). Left: experimental group; right: control group.

Figure 3 presents the evolution of the average α-DFA exponents during the four weeks of the experiment, in the solo and the duo sequences. Because the average α-DFA exponents for the experimenter were obtained from a single individual, the analysis of variance cannot be applied in the present case. These figures, however, suggest a close convergence of the mean exponents of the experimenter and those of the participants in the experimental group, over the 4 weeks. Note also that this convergence appears very early during the experiment, in the first week. This convergence appears less obvious in the control group. We present in Table 1 the average correlations between the α-DFA exponents of the participants and the corresponding exponents for the experimenter, computed over the 4 weeks, in the two groups. High correlations were observed in the experimental group, revealing a close statistical matching between the series simultaneously produced by the participants and the experimenter. The following analyses will check whether this statistical matching corresponds to a genuine complexity matching effect, or rather to a more local mode of synchronization. In contrast, correlations appeared moderate and extremely variable in the control group, suggesting a poor statistical matching between series.

**Table 1:** Average correlation between the α-DFA exponents of the participants and the corresponding exponents for the experimenter (standard deviations in brackets), computed over the 4 weeks of the experimental protocol.

	Week 1	Week 2	Week 3	Week 4
Experimental group	0.95 (0.05)	0.97 (0.04)	0.96 (0.05)	0.98 (0.01)
Control group	0.34 (0.44)	0.51 (0.30)	0.33 (0.39)	0.44 (0.33)

Interestingly, one could observe in Figure 3 that in the experimental condition, the experimenter seems poorly affected by synchronization. In contrast, participants appear strongly attracted toward the experimenter, as predicted by the complexity matching framework. In contrast, in the control condition the experimenter and the participants seem converging toward a median level of complexity, halfway between their solo levels.

**FIGURE 4:**
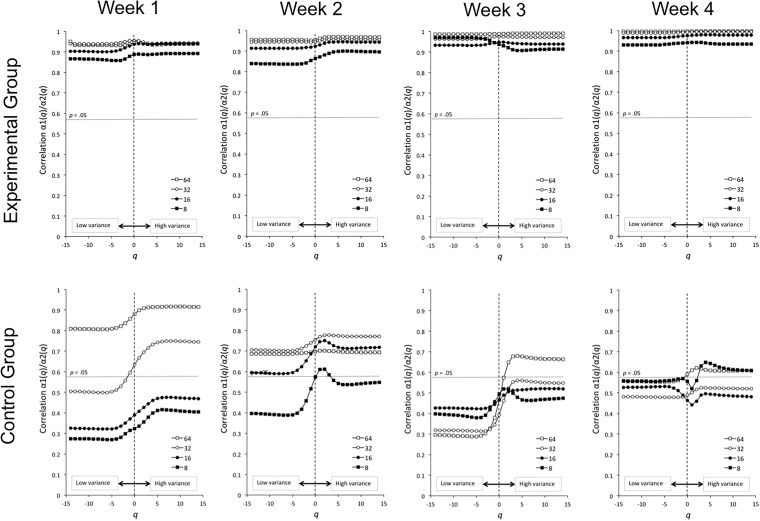
Correlation functions *r*(*q*), for the four ranges of intervals considered (8 to *N*/2, 16 to *N*/2, 32 to *N*/2, and 64 to *N*/2), for the experimental group (top row) and the control group (bottom row), and over the 4 weeks. *q* represents the set of orders over which the MF-DFA algorithm was applied.

Figure 4 presents the average correlation functions *r*(*q*) between multifractal spectra, for the two groups (top row: experimental; bottom row: control), and the 4 weeks. Correlation coefficients are plotted against their corresponding *q*-values. We displayed four correlation functions, according to the shortest interval length considered (i.e., 8, 16, 32, or 64). For the experimental group, the correlation functions are significant, whatever the considered range of intervals. This result suggests the presence of a complexity matching effect within the dyads (Delignières et al., 2016). Note that the complexity matching effect appears from the first week of the experiment, and tends to become stronger over weeks. In contrast, in the control group, the correlation functions exhibit lower, and often non-significant values, especially when the largest ranges of intervals are considered (i.e., 8 to *N*/2 and 16 to *N*/2).

**FIGURE 5:**
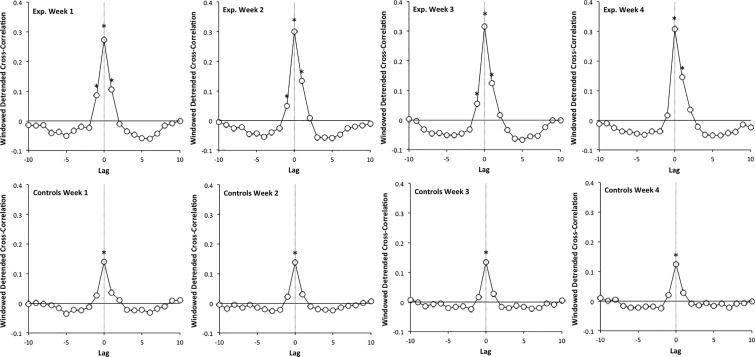
Averaged WDCC functions, from lag –10 to lag 10, for the experimental group (top row) and the control group (bottom row), and for the 4 weeks. Stars (^∗^) indicate coefficients significantly different from zero.

The averaged WDCC functions are reported in Figure 5, for the experimental group (top row) and the control group (bottom row), and for the 4 weeks. These functions systematically present a peak at lag 0, which appears higher for the experimental group (about 0.3) than for the control group (about 0.15). In both groups and all weeks, however, the location *t*-tests, comparing the obtained values to zero, are significant. The rather moderate values obtained in the experimental group are conformable to that previously obtained in similar experiments (Konvalinka et al., 2010; Delignières and Marmelat, 2014; Coey et al., 2016; Den Hartigh et al., 2018), and to that expected from a complexity matching synchronization (Almurad et al., 2017; Roume et al., 2018). These results provide evidence that synchronization, in this condition, is dominated by a complexity matching effect. In contrast, the values observed in the control group are very low, and suggest a quite poor, or just intermittent synchronization within dyads.

Another interesting indication is provided by the cross-correlation values at lag −1 and lag 1, which appear positive and significantly different from zero in the experimental group. This shows that synchronization, while clearly dominated by a complexity matching effect, also involves cycle-to-cycle discrete correction processes: both partners tend to (moderately) correct their current step duration on the basis of the asynchrony they perceived at the preceding heel-strike (see Roume et al., 2018, for a deeper analysis of WDCC properties). One could note a dissymmetry in these correction processes, the lag 1 values being higher than the lag −1 value: According to our conventions, this indicates that participants corrected his/her step duration to a greater extent than the experimenter did. Additionally this dissymmetry, negligible during the first week, becomes more and more salient over weeks. In contrast, we found no trace of correction processes in the control condition.

## Discussion

The three hypotheses that motivated this experimental work are validated:

(1) When an older person is invited to walk in synchrony, arm-in-arm, with a healthy partner, synchronization is mainly achieved through complexity matching. This hypothesis was validated by the two analysis methods we applied to the collected series: The correlation functions between multi-fractal spectra remained significant, whatever the range of exponents considered, revealing a global, multi-scale synchronization between series, and the WDCC functions exhibited a typical positive peak at lag 0, suggesting an immediate synchronization between systems. WDCC results showed that synchronization was clearly dominated by a complexity matching effect, even if slight cycle-to-cycle correction processes were also present, especially for participants, which tended to correct their steps on the basis of previous asynchronies. The main important result, at this level, was to show that forced synchronization, between systems of different levels of complexity, is based on similar processes than forced synchronization between systems of similar complexities (Almurad et al., 2017). Interestingly, the complexity matching effect appeared immediately, from the very first duo sequences, from the moment that the instruction of close synchrony with the experimenter was provided and respected.

(2) Considering two systems of different levels of complexity, complexity matching results in an attraction of the less complex system toward the more complex one. This result is one of the most interesting of this experiment, and clearly in line the theoretical conclusions afforded by Mahmoodi et al. (2018). The theoretical framework developed by these authors, highlighting the fundamental role of crucial events in the complexity matching effect, and the close relationship between crucial events and multifractality (see also Bohara et al., 2017; Mahmoodi et al., 2017), supports the emergence of stimulating hypotheses, concerning the true nature of the loss of complexity with age and disease, and the processes that underlie the restoration process observed in the present experiment. Note that one could also argue that a complex system being intrinsically more stable, this attraction results from the relative instability of the less complex one. However, the results in the control group (both systems being equally attracted by each other) seems contradicting this alternative explanation.

(3) A prolonged experience of complexity matching, between two systems of different levels of complexity, allows enhancing the complexity of the less complex system, this effect being persistent over time. In the context of our experiment this result suggests a possible restoration of complexity in older people. Note that we tested the persistence of this restoration through a unique post-test, performed 2 weeks after the end of the training sessions. Further investigations are necessary for analyzing the persistence of this effect, its probable decay over time, and the effects of an additional training session when a significant decay is observed (one could hypothesize that restoration could occur more quickly during a second administration of the rehabilitation protocol).

As far as we know, this is the first evidence for a possible restoration of complexity in deficient systems. Recently Warlop et al. (2017) evoked the effects of Nordic Walking for restoring complexity in patients suffering from Parkinson’s disease, but their experiment focused essentially on the immediate effects of the adoption of a specific locomotion pattern, rather than on the long-term effects of a rehabilitation protocol.

In this experiment a statistical effect was obtained at the beginning of the fourth week. During a pre-testing period, we tried to pursue training up to the obtaining of an increase of long-range correlations. We systematically obtained this effect at the beginning of the fourth week, and decided to limit the protocol to four successive weeks. However, the analysis of individual results shows that this restoration could occur early, at the beginning of the third week. The most important observation is that complexity matching does not spontaneously induce a restoration of complexity in solo sequences, and a repeated and prolonged experience of complexity matching seems necessary. The results of the control group show that an intense training in walking is not sufficient. Walking in close synchrony with a healthy partner appears a key factor in the restoration process, and our analyses about the duo sequences suggest that complexity matching may be the essential ingredient.

Some limitations of the present study have to be pointed out. First, it should be noticed that we evidence in this experiment the possibility of a restoration of complexity, and we just *suppose*, on the basis on previous assumptions, that this should result in a more adaptable and stable locomotion, and a decrease of fall propensity. Longitudinal studies, using clinical tests and systematic follow-up survey, should be necessary for confirming this hypothesis. However, this was clearly beyond the scope of the present work.

Second, this experiment was extremely difficult to organize (due to the availability of the indoor track), and was very challenging for both the participants and the experimenter. It took up to 14 months for performing the whole protocol for the 24 participants. For practical reasons, we decided to design the protocol with a single experimenter, who performed all sequences with all participants. This had the advantage of standardizing the experimental conditions, but introduced a possible bias, as our results could be related to some hidden and unsuspected qualities of this specific person. It seems obviously necessary to replicate these results with other accompanying persons. A second experiment is currently engaged in our laboratory for clarifying this point.

Finally, considering the intrinsic difficulty of the experimental protocol, we recruited participants that presented a normal, non-pathological aging, and consequently a rather moderate loss of complexity. The average DFA exponent characterizing the step duration series of our participants was of about 0.83, clearly higher than the mean value reported by Hausdorff et al. (1997) in their group of elderly participants (0.68). Further investigations are required for adapting and testing this kind of protocol with patients suffering of more pronounced locomotion diseases and greater losses of complexity.

## Conclusion

In conclusion, this experiment should not be considered a clinical study, aiming at validating and promoting a rehabilitation strategy, but rather a fundamental work testing a theoretical hypothesis (the restoration of complexity in living organisms through complexity matching). We hope, obviously, that it could inspire clinicians for developing, validating and diffusing effective rehabilitation protocols. Currently most research in locomotion rehabilitation focuses on sophisticated devices, involving virtual reality, metronomic guidance, robotic assistance, etc. We are not sure, however, that genuine complexity matching could occur in the interaction with an artificial device (Delignières and Marmelat, 2014). Our experiment suggests that rehabilitation could be achieved with simpler, less expensive and also more humane means. We think especially to countries and situations where the access to sophisticated medical care remains difficult, and often unconceivable. We would be proud that our work can give scientific support to this simple prescription: “Take your eldest’s arm and walk together.”

## Author Contributions

ZA and DD contributed to conception and design of the study and wrote the first draft of the manuscript. CR developed the measuring device. ZA conducted the experiment and performed the statistical analysis. DD and HB supervised the whole project. All authors contributed to manuscript revision, and read and approved the submitted version.

## Conflict of Interest Statement

The authors declare that the research was conducted in the absence of any commercial or financial relationships that could be construed as a potential conflict of interest.

## References

[B1] Abney D. H. Paxton A. Dale R. Kello C. T. (2014). Complexity matching in dyadic conversation. *J. Exp. Psychol. Gen.* 143 2304–2315. 10.1037/xge0000021 25285431

[B2] Almurad Z. M. H. Delignières D. (2016). Evenly spacing in detrended fluctuation analysis. *Phys. Stat. Mech. Its Appl.* 451 63–69. 10.1016/j.physa.2015.12.155 28750259

[B3] Almurad Z. M. H. Roume C. Delignières D. (2017). Complexity matching in side-by-side walking. *Hum. Mov. Sci.* 54 125–136. 10.1016/j.humov.2017.04.008 28460275

[B4] Aquino G. Bologna M. West B. J. Grigolini P. (2011). Transmission of information between complex systems: 1/f resonance. *Phys. Rev. E Stat. Nonlin. Soft Matter Phys.* 83:051130. 10.1103/PhysRevE.83.051130 21728513

[B5] Bohara G. Lambert D. West B. J. Grigolini P. (2017). Crucial events, randomness, and multifractality in heartbeats. *Phys. Rev.* E 96:062216 10.1103/PhysRevE.96.062216 29347370

[B6] Coey C. A. Washburn A. Hassebrock J. Richardson M. J. (2016). Complexity matching effects in bimanual and interpersonal syncopated finger tapping. *Neurosci. Lett.* 616 204–210. 10.1016/j.neulet.2016.01.066 26840612 PMC4810785

[B7] Delignières D. Almurad Z. M. H. Roume C. Marmelat V. (2016). Multifractal signatures of complexity matching. *Exp. Brain Res.* 234 2773–2785. 10.1007/s00221-016-4679-4 27225255

[B8] Delignières D. Marmelat V. (2012). Fractal fluctuations and complexity: current debates and future challenges. *Crit. Rev. Biomed. Eng.* 40 485–500. 10.1615/CritRevBiomedEng.2013006727 23356693

[B9] Delignières D. Marmelat V. (2014). Strong anticipation and long-range cross-correlation: application of detrended cross-correlation analysis to human behavioral data. *Phys. Stat. Mech. Its Appl.* 394 47–60. 10.1016/j.physa.2013.09.037

[B10] Delignieres D. Ramdani S. Lemoine L. Torre K. Fortes M. Ninot G. (2006). Fractal analyses for “short” time series: a re-assessment of classical methods. *J. Math. Psychol.* 50 525–544. 10.1016/j.jmp.2006.07.004

[B11] Den Hartigh R. J. R. Marmelat V. Cox R. F. A. (2018). Multiscale coordination between athletes: complexity matching in ergometer rowing. *Hum. Mov. Sci.* 57 434–441. 10.1016/j.humov.2017.10.006 29107321

[B12] Fine J. M. Likens A. D. Amazeen E. L. Amazeen P. G. (2015). Emergent complexity matching in interpersonal coordination: local dynamics and global variability. *J. Exp. Psychol. Hum. Percept. Perform.* 41 723–737. 10.1037/xhp0000046 25798782

[B13] Goldberger A. L. Amaral L. A. N. Hausdorff J. M. Ivanov P. C. Peng C. K. Stanley H. E. (2002a). Fractal dynamics in physiology: alterations with disease and aging. *Proc. Natl. Acad. Sci. U S A.* 99 2466–2472. 11875196 10.1073/pnas.012579499 PMC128562

[B14] Goldberger A. L. Peng C. K. Lipsitz L. A. (2002b). What is physiologic complexity and how does it change with aging and disease?. *Neurobiol. Aging* 23 23–26. 10.1016/S0197-4580(01)00266-4 11755014

[B15] Hausdorff J. M. Ashkenazy Y. Peng C. K. Ivanov P. C. Stanley H. E. Goldberger A. L. (2001). When human walking becomes random walking: fractal analysis and modeling of gait rhythm fluctuations. *Physica A* 302 138–147. 10.1016/S0378-4371(01)00460 12033228

[B16] Hausdorff J. M. Mitchell S. L. Firtion R. Peng C. K. Cudkowicz M. E. Wei J. Y. (1997). Altered fractal dynamics of gait: reduced stride-interval correlations with aging and Huntington’s disease. *J. Appl. Physiol.* 82 262–269. 10.1152/jappl.1997.82.1.262 9029225

[B17] Hausdorff J. M. Peng C. Ladin Z. Wei J. Goldberger A. (1995). Is walking a random-walk - evidence for long-range correlations in stride interval of human gait. *J. Appl. Physiol.* 78 349–358. 10.1152/jappl.1995.78.1.349 7713836

[B18] Hausdorff J. M. Purdon P. L. Peng C. K. Ladin Z. Wei J. Y. Goldberger A. L. (1996). Fractal dynamics of human gait: stability of long-range correlations in stride interval fluctuations. *J. Appl. Physiol.* 80 1448–1457. 10.1152/jappl.1996.80.5.1448 8727526

[B19] Kantelhardt J. W. Zschiegner S. A. Koscielny-Bunde E. Havlin S. Bunde A. Stanley H. E. (2002). Multifractal detrended fluctuation analysis of nonstationary time series. *Phys. A Stat. Mech. Its Appl* 316 87–114. 10.1016/S0378-4371(02)01383

[B20] Konvalinka I. Vuust P. Roepstorff A. Frith C. D. (2010). Follow you, follow me: continuous mutual prediction and adaptation in joint tapping. *Q. J. Exp. Psychol.* 63 2220–2230. 10.1080/17470218.2010.497843 20694920

[B21] Lipsitz L. Goldberger A. (1992). Loss of complexity and aging-potential applications of fractals and chaos theory to senescence. *Jama.* 267 1806–1809. 10.1001/jama.267.13.1806 1482430

[B22] Mahmoodi K. West B. J. Grigolini P. (2017). *On The Dynamical Foundation of Multifractality.* Available at: http://arxiv.org/abs/1707.05988 [accessed February 9, 2018].

[B23] Mahmoodi K. West B. J. Grigolini P. (2018). *Complexity Matching and Requisite Variety.* Available at: http://arxiv.org/abs/1806.08808 [accessed October 30, 2018].

[B24] Marmelat V. Delignières D. (2012). Strong anticipation: complexity matching in interpersonal coordination. *Exp. Brain Res.* 222 137–148. 10.1007/s00221-012-3202 22865163

[B25] Marmelat V. Delignières D. Torre K. Beek P. J. Daffertshofer A. (2014). “Human paced” walking: followers adopt stride time dynamics of leaders. *Neurosci. Lett.* 564 67–71. 10.1016/j.neulet.2014.02.010 24548624

[B26] Peng C. K. Buldyrev S. V. Havlin S. Simons M. Stanley H. E. Goldberger A. L. (1994). Mosaic organization of dna nucleotides. *Phys. Rev. E* 49 1685–1689. 10.1103/PhysRevE.49.1685 9961383

[B27] Peng C. K. Havlin S. Stanley H. E. Goldberger A. L. (1995). Quantification of scaling exponents and crossover phenomena in nonstationary heartbeat time-series. *Chaos* 5 82–87. 10.1063/1.166141 11538314

[B28] Podobnik B. Stanley H. E. (2008). Detrended cross-correlation analysis: a new method for analyzing two nonstationary time series. *Phys. Rev. Lett.* 100:084102. 10.1103/PhysRevLett.100.084102 18352624

[B29] Roume C. Almurad Z. M. H. Scotti M. Ezzina S. Blain H. Delignières D. (2018). Windowed detrended cross-correlation analysis of synchronization processes. *Phys. A Stat. Mech. Its Appl.* 503 1131–1150. 10.1016/j.physa.2018.08.074

[B30] Sleimen-Malkoun R. Temprado J.-J. Hong S. L. (2014). Aging induced loss of complexity and dedifferentiation: consequences for coordination dynamics within and between brain, muscular and behavioral levels. *Front. Aging Neurosci.* 6:140. 10.3389/fnagi.2014.00140 25018731 PMC4073624

[B31] Stephen D. G. Dixon J. A. (2011). Strong anticipation: multifractal cascade dynamics modulate scaling in synchronization behaviors. *Chaos Solitons Fractals* 44 160–168. 10.1016/j.chaos.2011.01.005

[B32] Stephen D. G. Stepp N. Dixon J. A. Turvey M. T. (2008). Strong anticipation: sensitivity to long-range correlations in synchronization behavior. *Phys. Stat. Mech. Its Appl.* 387 5271–5278. 10.1016/j.physa.2008.05.015

[B33] Torre K. Varlet M. Marmelat V. (2013). Predicting the biological variability of environmental rhythms: weak or strong anticipation for sensorimotor synchronization?. *Brain Cogn.* 83 342–350. 10.1016/j.bandc.2013.10.002 24212115

[B34] Vaillancourt D. E. Newell K. M. (2002). Changing complexity in human behavior and physiology through aging and disease. *Neurobiol. Aging* 23 1–11. 10.1016/S0197-4580(01)00247-0 11755010

[B35] Van Orden G. C. Moreno M. A. Holden J. G. (2003). A proper metaphysics for cognitive performance. *Nonlin. Dyn. Psychol. Life Sci.* 7 49–60. 10.1023/A:1020462025387 12876446

[B36] Warlop T. Detrembleur C. Buxes Lopez M. Stoquart G. Lejeune T. Jeanjean A. (2017). Does nordic walking restore the temporal organization of gait variability in Parkinson’s disease. *J. NeuroEng. Rehabil.* 14:17. 10.1186/s12984-017-0226-1 28222810 PMC5320697

[B37] West B. J. Geneston E. L. Grigolini P. (2008). Maximizing information exchange between complex networks. *Phys. Rep.* 468 1–99. 10.1016/j.physrep.2008.06.003

